# Systematic review of the efficacy and safety of biologically and tissue-derived therapies for the treatment of diabetic foot

**DOI:** 10.3389/fendo.2026.1800592

**Published:** 2026-04-07

**Authors:** Jia Teng, Fuyang Lin, Ling Wang, Mingjun Jiang, Zhiqiao Li, Guanchi Yan, Jia Mi

**Affiliations:** 1 College of Traditional Chinese Medicine, Changchun University of Chinese Medicine, Changchun, China; 2 The Affiliated Hospital to Changchun University of Chinese Medicine, Changchun University of Chinese Medicine, Changchun, China

**Keywords:** autologous cell therapy, autologous skin transplantation, diabetic foot, meta-analysis, platelet-rich plasma, systematic review

## Abstract

**Background:**

Diabetic foot ulcers are a common and serious complication in diabetic patients, and traditional treatments have limited healing rates. Autologous cells and related products, as an emerging therapy, require systematic evaluation for their efficacy and safety.

**Objective:**

To systematically evaluate and meta-analyze the efficacy and safety of autologous cells and related products in the treatment of diabetic foot ulcers.

**Methods:**

Relevant randomized controlled trials up to November 2025 were searched in PubMed and Embase databases. Patients with diabetic foot ulcers were included, and the intervention was autologous cell therapy. Data on healing rate, percentage reduction in ulcer area, and healing days were extracted and meta-analyzed using RevMan 5.3.

**Results:**

A total of 26 randomized controlled trials (RCTs) involving 2,214 patients were included. The autologous cell therapy group showed a significantly higher rate of complete healing than the control group (RR = 0.54, 95% CI: 0.45). The difference in ulcer area reduction was significantly greater (MD = 24.6%, 95%CI: 18.3-30.9, P<0.001) and healing time was significantly shorter (MD= -48.8 days, 95% CI: -74.19 to -23.48, P = 0.0002). There were no significant differences in adverse events such as amputation and mortality between the two groups. In conclusion, autologous cell therapy can significantly improve the healing rate and accelerate ulcer healing in diabetic foot ulcers, with good safety, making it a promising treatment option.

**Systematic review registration:**

https://www.crd.york.ac.uk/prospero/, identifier CRD420261293843.

## Introduction

Diabetic foot ulcers are a common and serious complication of late-stage diabetes, affecting approximately 15% of diabetic patients, with 15% of these cases leading to amputation (1). Diabetes-related foot disease is one of the most serious complications of diabetes and a major source of reduced quality of life and economic burden for patients (2). With the increasing incidence of diabetes in adolescents, the incidence of diabetic foot ulcers (DFUs) is also becoming increasingly younger (3, 4). Common Western medical treatments for diabetic foot include tissue excision, drainage of abscesses or infected chambers, resection of necrotic or infected bone, or vascular reconstruction (5). The goal of the vascular reconstruction procedure is to restore blood flow to at least one artery in the foot (2). Traditional treatments still rely on surgical debridement (6), antibacterial dressings (7), and flap transplantation (8). While effective, these methods often carry surgical risks. In recent years, autologous cell therapy has gained attention due to its readily available source, low risk of immune rejection, and high potential for promoting tissue regeneration. These therapies encompass a diverse range of products, including but not limited to, autologous cell therapies (e.g., hematopoietic stem cells, bone marrow stromal cells), platelet-rich plasma (PRP), and various skin substitutes (e.g., dehydrated human amniotic membrane, human placental-derived cells, acellular dermal matrices). Hematopoietic stem cells and bone marrow stromal cells (MSCs) are considered potential candidate cells. These cells contribute to the replacement of damaged endothelial cells and the formation of new blood vessels or vascular remodeling. Several autologous cell products have already been tested in randomized controlled trials and are gradually being applied clinically. For example, REGRANEX gel, the first FDA-approved product containing rhPDGF-BB, has been safely and effectively used to promote the healing of these skin wounds for over 25 years (9). Due to the fact that diabetic foot ulcers are often accompanied by complex conditions such as infection and ischemia, designing rigorous Phase III trials is extremely difficult, resulting in a lack of high-quality evidence from large-scale clinical trials, therefore, there are very few cell therapies approved by the FDA and EMA. Other treatments include autologous platelet-rich plasma, dehydrated human amniotic membrane, and human placental-derived cells. However, their efficacy and safety require further systematic evaluation. In this review, we aimed to evaluate the efficacy of autologous cell therapy and related products in patients with diabetic foot. All included patients had diabetic foot. We also analyzed secondary outcome measures such as percentage reduction in ulcer area and healing days, and performed subgroup analyses based on country, age, glycated hemoglobin, and duration of diabetic foot disease.

## Methods

This systematic review was conducted in accordance with the Priority Reporting Program for Systematic Reviews and Meta-analyses (PRISMA) guidelines. The primary research questions focused on the efficacy of autologous cell and related treatments in achieving complete healing rate, healing time, and treatment duration in diabetic foot ulcers compared to standard treatments or non-autologous products. The primary outcome measure was complete healing rate, and secondary outcome measures included percentage reduction in ulcer area and days to healing.

### Registration

This systematic review was conducted in accordance with the Preferred Reporting Items for Systematic Reviews and Meta-Analyses (PRISMA) guidelines. The study protocol was registered on the International Prospective Register of Systematic Reviews (PROSPERO) with the registration number CRD420261293843. Any amendments to the protocol during the review process are documented and justified in the relevant sections.

### Ethics

This meta-analysis was not subject to ethical review because it used previously published data and no new data was collected. No one’s consent was obtained to participate in this study, nor was approval from an institutional review board required.

### Inclusion and exclusion criteria

Studies meeting the following criteria were included:

Study type: The clinical trial type is a randomized controlled trial (RCT), and it is published in English;

Study subjects: Patients diagnosed with diabetic foot;

Intervention: The intervention group received any form of autologous cell therapy and related treatments;

Control measures: The control group received either placebo or standard treatment. Placebo included, but was not limited to, normal saline, diluted autologous peripheral blood (PB), and matrix of cell products;

Outcome measures: The primary outcome measure was complete healing rate, and the secondary outcome measures were the percentage reduction in ulcer area and healing time.

Studies meeting the following criteria were excluded:

Non- randomized controlled trial studies;

Skin ulcers or slow-healing wounds not caused by diabetes;

The following data were not reported: complete healing rate, percentage reduction in ulcer area, healing time, treatment time, and adverse events;

Data is incomplete or cannot be extracted;

Duplicate publications, conference abstracts, or review articles;

### Data sources and retrieval strategies

The system searches using PubMed and Embase databases, covering the period from database creation to November 2025. Search terms include: Diabetic Foot [title/abstract], skin substitute [title/abstract], randomly [title/abstract], clinical trials [title/abstract], and randomized controlled trial [title/abstract], etc.

### Quality assessment

The quality of the included studies was assessed using the Cochrane Risk of Bias Assessment Tool. This scale assessed the adequacy of random sequence generation, concealment of allocation sequences, blinding of subjects and assessors, blinding of outcome assessment, completeness of outcome data, selectivity of outcome reporting, and other potential biases.

### Data extraction

Information extracted from included randomized controlled trials (RCTs) is as follows: country and center, study design type, patient baseline characteristics, type and dose of cell therapy in the experimental group, and outcome endpoints. The primary outcome endpoint was complete healing rate. Secondary outcome endpoints included percentage reduction in ulcer area and healing time. Data were summarized in graphical form using PowerPoint software.

### Quality evaluation

Data were extracted independently by two researchers and included first author, publication year, sample size, intervention, control, baseline characteristics, and outcome measures. Safety was described by amputation or death. Study quality was assessed using the Cochrane risk of bias assessment tool.

### Statistical analysis

Meta-analysis was performed using RevMan 5.3. Hazard ratios were used for binary variables, and mean differences were used for continuous variables. 95% confidence intervals were calculated. Heterogeneity was assessed using the I² statistic; if I² > 50%, a random-effects model was used. Subgroup analysis was performed using RevMan 5.3, and sensitivity analysis was conducted using R language 4.4.2.

Analysis was performed using RevMan 5.3 software. Continuous data are presented as mean ± standard deviation. If data are presented as median or quartiles, the mean and standard deviation were estimated. In cases of non-compliance and missing results during the study, the intention-to-treat principle was followed. Due to the inherent clinical and methodological heterogeneity of included studies, all meta-analyses employed random-effects models, regardless of the presence of statistical heterogeneity. The Mantel-Haenszel method was used to pool results. I² statistic, Q test, and χ² test were used to assess heterogeneity among studies.

### Publication bias

Based on the included country, mean patient age (≤ 60 years vs> 60 years), glycated hemoglobin (< 8.0% vs ≥ 8.0%), and disease duration (≤ 20 weeks vs> 20 weeks). Sensitivity analysis was performed by comparing the differences between random-effects and fixed-effects models. Publication bias was examined using a funnel plot.

## Result

### Research characteristics

**Figure 1:**
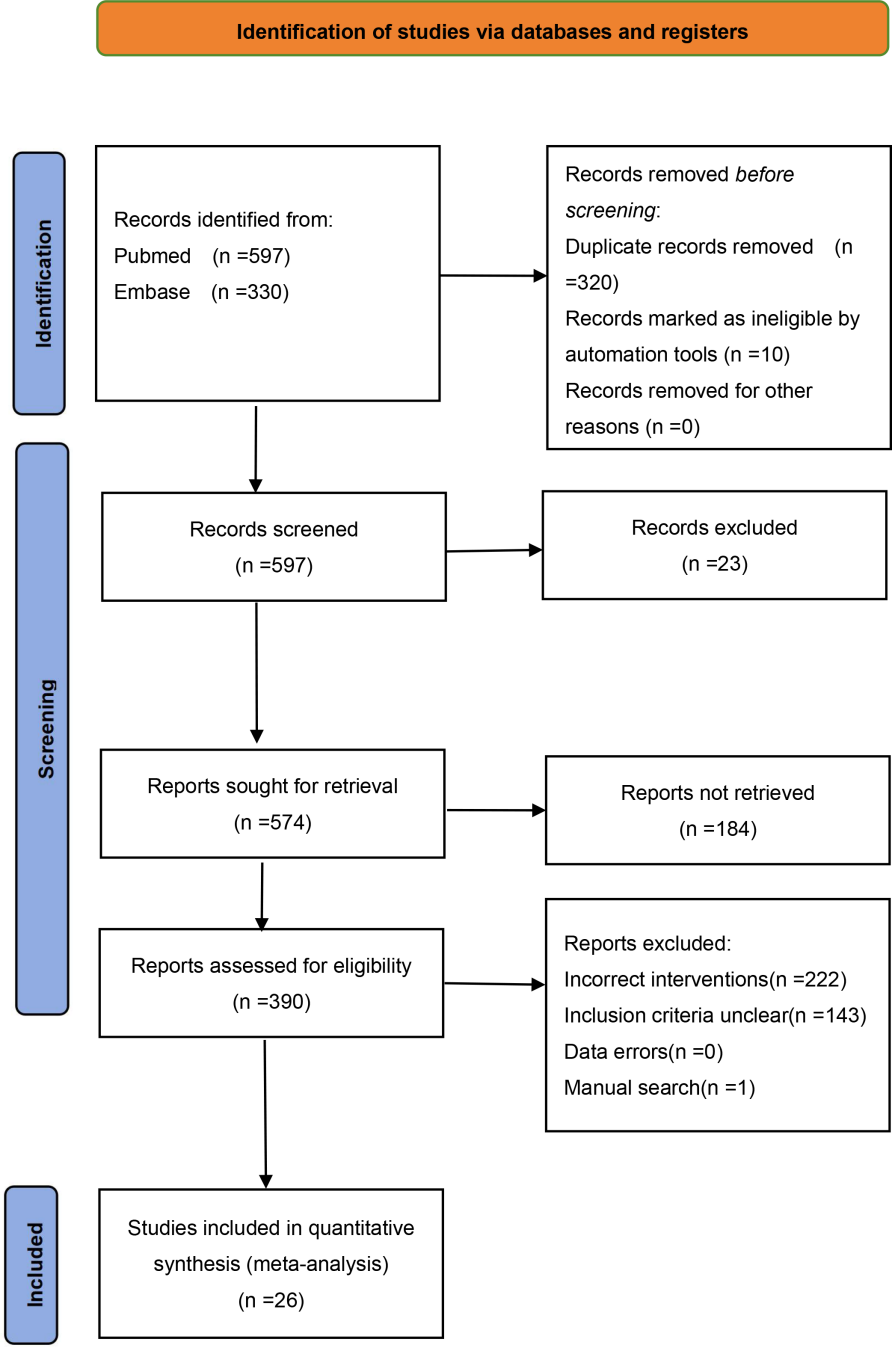
PRISMA flow diagram of the study selection process.

The flowchart of the included studies is shown in **Figure 1** (10–35). A preliminary search yielded a total of 927 articles, and after screening, 25 randomized controlled trials (RCTs) were included, among which were phase II and III clinical trials. One additional article was manually added based on its content, bringing the total to 26 RCTs, involving 2214 patients with diabetic foot. The included studies were published between 2013 and 2025, mostly multicenter studies with sample sizes ranging from 19 to 155 patients. Patients came from Europe, Asia, North America, and Oceania. The mean age of the included patients ranged from 58.2 to 75 years. Characteristics of the relevant studies are shown in **Table 1**.

**Table 1:** Baseline characteristics of the included studies.

First author (year)	National and center	Mean age (year) (± standard deviation) of the experimental group	Mean glycated hemoglobin in the experimental group (%) (± standard deviation)	Ulcer course in the experimental group (weeks) (± standard deviation)	The average ulcer area in the experimental group (cm²) (± standard deviation).	Experimental group sample size	Intervention methods in the experimental group	Follow-up period (months)
Tettelbach, W.(2019) (10)	US multicenter	57.4	7.8	20.8	3.2	54	Use dehydrated human amniotic membrane/chorionic membrane (dHACM)	4.0
Armstrong, DG (2023) (11)	US multicenter	60.1	7.4	22.6	3.5	50	Novel autologous heterogeneous skin constructs (AHSC)	NA
Ananian, CE (2018) (12)	US multicenter	55.3	8.7	28.5	7.2	38	Cryopreserved human placental membranes (vCPM)	NA
Tettelbach, W. (2019) (13)	US multicenter	58.3	8.0	20.5	2.6	101	Dehydrated human umbilical cord allogeneic graft (EpiCord)	1.0
Charles M Zelen (2013) (15)	US multicenter	55.0	NA	10.0	2.0	13	Dehydrated human amniotic membrane product (EpiFix) combined with standard care	1.5
Charles M Zelen (2014) (16)	US multicenter	60.8 ± 10.9	8.7 ± 2.2	11.0	1.4	20	Receive dehydrated human amniotic membrane/chorionic membrane transplantation (dHACM) once a week.	NA
Charles M Zelen (2017) (17)	US multicenter	61.5	7.9	NA	2.7	20	Cell-free reticular allogeneic human dermis (HR-ADM) plus standard treatment	NA
Sylvaine Clave (2025) (18)	French single center	68.1	NA	NA	NA	48	Standardized font platelet gel (RegenWound gel, RWG)	NA
Shawn Cazzell (2017) (19)	US multicenter	59.1	8.4	36.4	3.9	71	Human acellular dermal matrix (ADMs)	3.0
Thomas E Serena (2020) (20)	US multicenter	59.2	NA	NA	3.1	38	HSAM and Standard of Occupation (SOC)	1.0
Charles M Zelen (2018) (21)	US multicenter	55.0	7.8	NA	3.2	40	Aseptically processed decellularized reticular allogeneic human dermis (HR-ADM) combined with SOC	NA
Juan Xie (2020) (22)	China Single Center	60.5	7.8	21.6	11.8	25	Autologous platelet-rich gel (APG)	NA
Sarah Onida (2025) (23)	UK multicenter	67.6 ± 15.5	NA	20.0	11.1	36	Acellular dermal (DCD) allogeneic grafts	12.0
Shawn M Cazzell (2024) (24)	US multicenter	61.6	NA	12.0	4.3	109	Dehydrated amniotic chorionic membrane (dACM) combined with standard care (SoC)	NA
Robert Snyder (2024) (25)	US multicenter	58.3 ± 8.8	8.3 ± 1.62	78.8	5.1	59	Autologous whole blood clot (AWBC) combined with standard of care (SoC)	0.5
Alexander M Reyzelman (2025) (26)	US multicenter	57.7 ± 9.2	NA	10.1	3.6	46	Full-thickness acellular placental membrane allogeneic graft (FT-DPM) combined with standard therapy (SoC)	NA
Richard Pollak (2025) (28)	US multicenter	56.4	NA	NA	2.1	47	3×10^6^ human placental-derived cells (PDA-002)	NA
Lisa J Gould (2022) (29)	US multicenter	59.4 ± 13.2	8.1 ± 1.65	15.2 ± 10.4	3.1 ± 3.4	50	Microvascular tissue allografts (PMVT)	NA
David G Armstrong (2022) (14)	US multicenter	61.2	7.1	17.5	4.2	50	Bioactive cross-sectional epidermal allografts (BSA)	NA
Hussam Alhawari (2023) (30)	US multicenter	NA	8.5	27.6	13.9	10	Human platelet lysate (hPL)	NA
Hyung Min Hahn (2021) (31)	South Korea Single Center	63.5 ± 12.9	7.1 ± 1.8	NA	16.3 ± 10.3	15	Ready-to-use micronized human acellular dermal matrix (MHADM) combined with conventional NPWT	6.0
Lawrence A Lavery (2014) (32)	US multicenter	55.5	8.0	16.4	3.4	50	Grafix treatment	3.0
Charles M Zelen (2016) (33)	US multicenter	63.3	7.5	17.3	1.7	32	dHACM	NA
Lawrence A DiDomenico (2018) (34)	US multicenter	60.1	7.6	NA	2.1	40	dHACA + Standard Treatment (SOC) group	NA
Laurens Manning (2022) (35)	Australian multicenter	61.5 ± 14.3	NA	NA	11.0	24	Spray-on autologous skin grafting (ReCell)	NA
Xue-Qin Wang (2024) (27)	China Multicenter	63.26 ± 10.94	8 ± 1.52	NA	NA	38	Autologous platelet-rich gel (APG)	6.0

### Quality assessment

**Figure 2:**
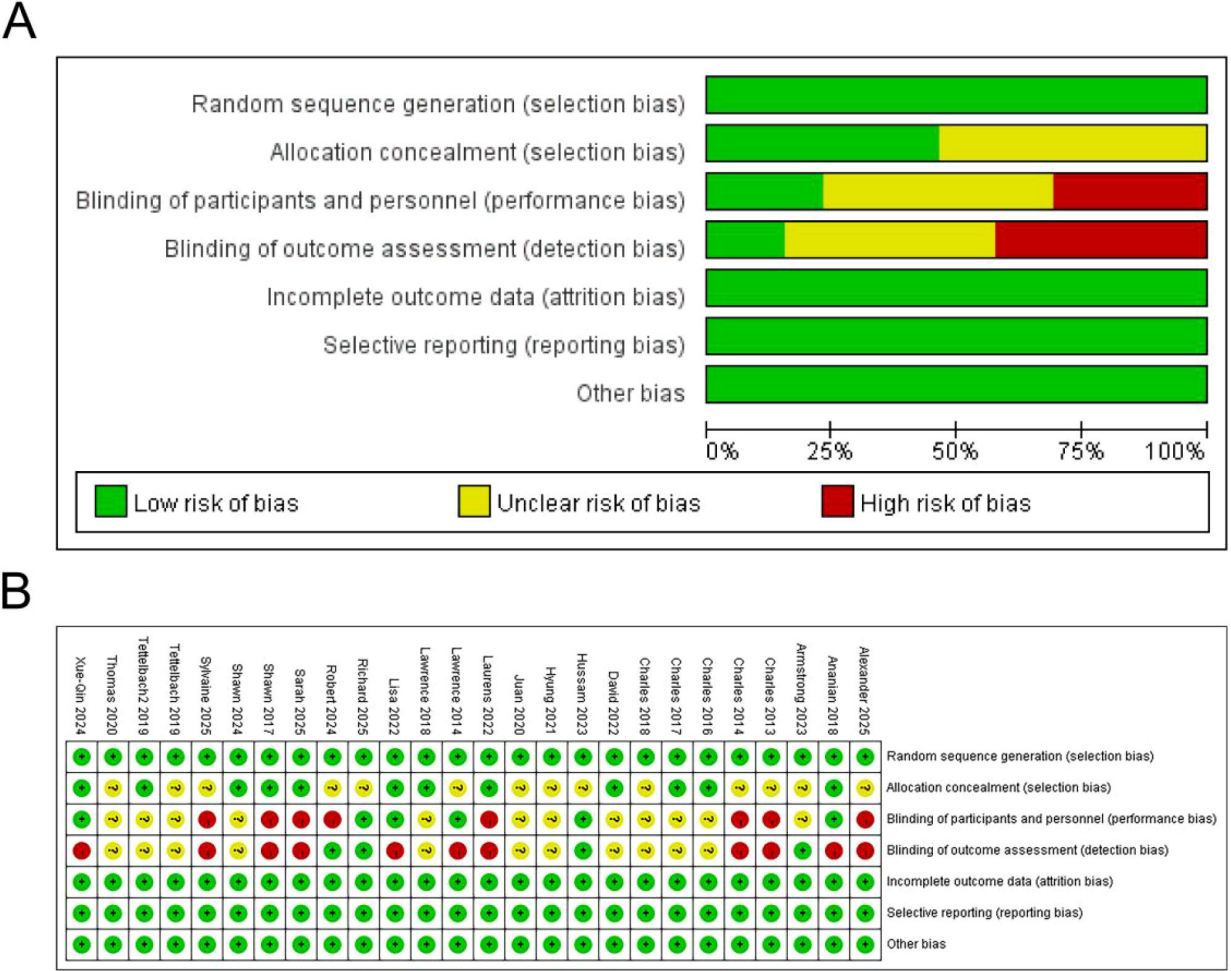
Risk of bias summary. **(A)** Risk of bias graph: review authors’ judgements about each risk of bias item presented as percentages across all included studies. **(B)** Risk of bias summary: review authors’ judgements about each risk of bias item for each included study.

The risk of bias was assessed using the Cochrane tool, and the results are shown in **Figures 2A, B**. Overall, the quality of the included studies was moderate. Most studies were considered to have a low risk of bias in terms of random sequence generation and incomplete outcome data. A considerable number of studies did not mention details of allocation concealment. Some studies lacked blinding of participants and personnel or blinding of outcome assessment. Nevertheless, all studies reported the primary outcome measure of complete healing rate, ensuring the reliability of the core conclusions of this meta-analysis.

### Primary outcome measure

#### Complete healing rate

**Figure 3:**
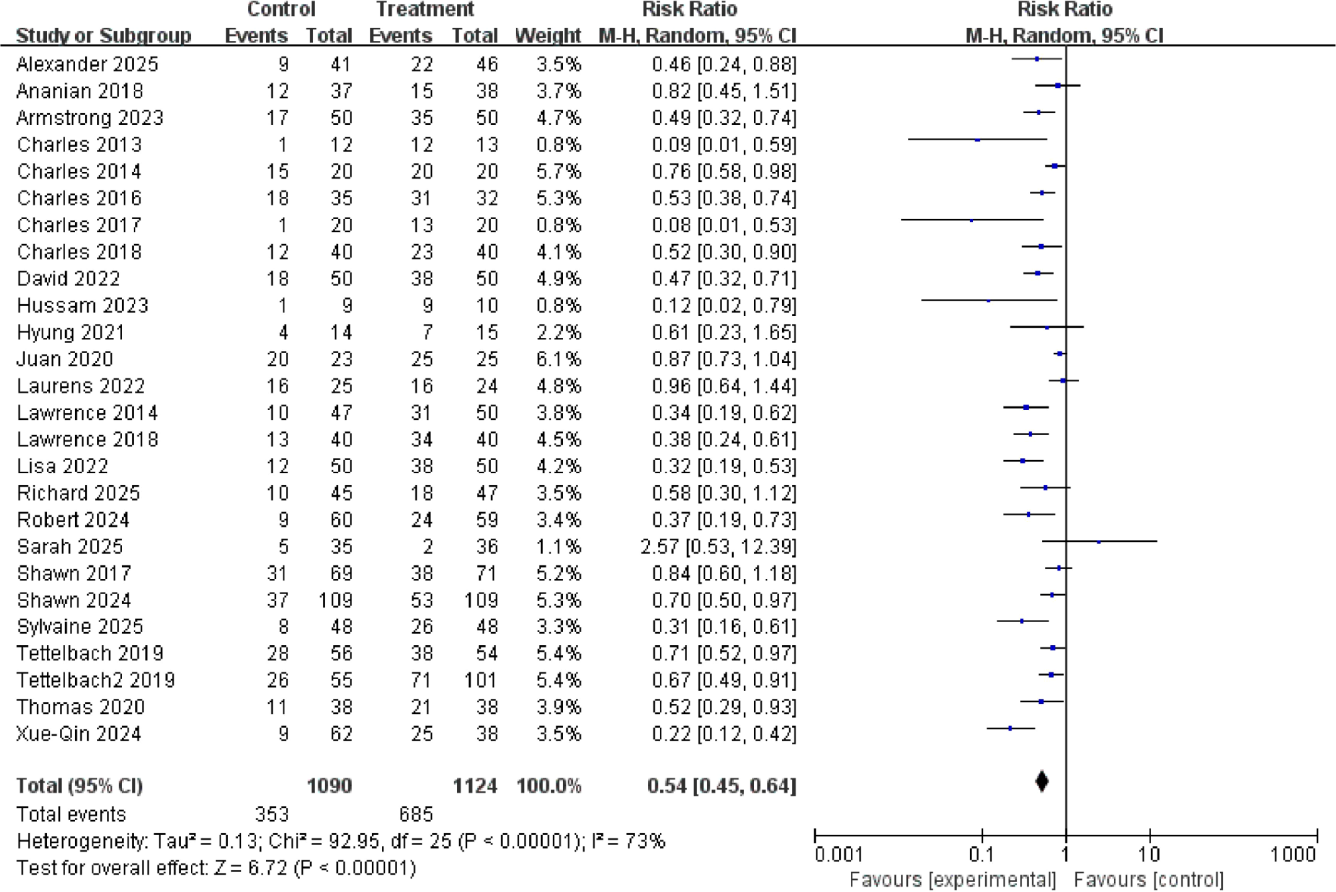
Forest plot of complete healing rate.

26 randomized controlled trials, involving a total of 2,214 participants, reported complete cure rates. Results showed a significantly higher healing rate in the treatment group compared to the control group (relative risk, 0.54; 95% confidence interval [CI], 0.45–0.64; P < 0.00001), indicating that autologous cell therapy was approximately 1.85 times more likely to achieve complete healing of diabetic foot ulcers than the control group (1/0.54 ≈ 1.85), as shown in **Figure 3**. This clearly and strongly confirms that the meta-analysis conclusions regarding the significant improvement in diabetic foot ulcer healing rates (RR ≈ 1.9) by this intervention are robust and reliable, unaffected by any single study.

### Secondary outcome measure

#### Percentage reduction in ulcer area

**Figure 4:**
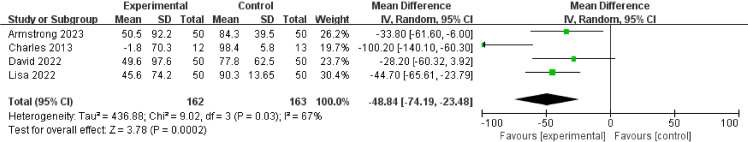
Forest plot of percentage reduction in ulcer area.

Four randomized controlled trials were conducted, with a total of 325 participants reporting the percentage reduction in ulcer area. Results showed that the autologous cell therapy group significantly reduced the percentage of ulcer area (mean difference [MD], -48.8; 95% CI, -74.19 to -23.48; P = 0.0002), as shown in **Figure 4**. This means that, on average, autologous cell therapy reduced ulcer area by approximately one-quarter more during the follow-up period, a difference that was clinically and statistically significant.

#### Healing time

**Figure 5:**
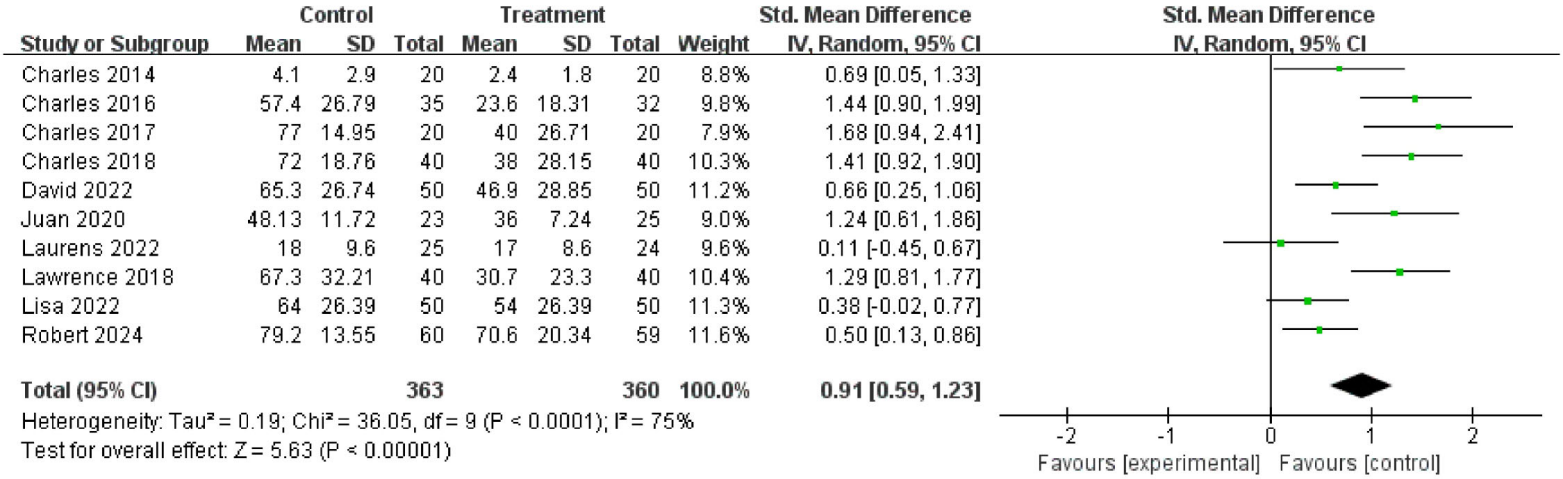
Forest plot of healing time.

Nine randomized controlled trials, involving 723 participants, reported healing days and standard deviations. The results showed that the efficacy of the experimental group was significantly superior to that of the control group (Z = 5.63, P < 0.00001). The meta-analysis conclusions regarding the intervention’s promotion of diabetic foot ulcer healing were highly robust. The exclusion of individual studies did not have a meaningful impact on the pooled effect, enhancing the reliability of the final conclusions. The results are shown in **Figure 5**.

### Safety outcome

Some studies reported mild local reactions, such as erythema, itching, or transient discomfort at the treatment site, but no serious systemic adverse events were reported. This indicates that autologous cell therapy and related products have a good safety profile and do not introduce additional serious risks.

### Sensitivity analysis

**Figure 6:**
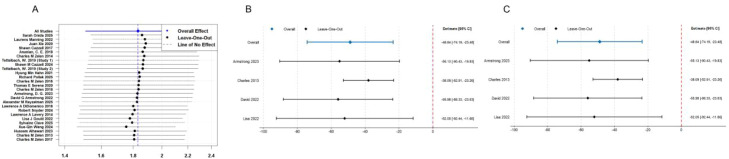
Sensitivity analysis.

All results obtained from the fixed model showed no significant difference from those obtained from the random model. The results demonstrate robustness. The results are shown in **Figure 6**.

### Subgroup analysis

To explore potential sources of heterogeneity and assess the consistency of treatment effects across different patient groups, we conducted pre-specified subgroup analyses based on the following factors: (1) country/region (to examine geographical or healthcare system differences); (2) mean age (≤60 years vs. >60 years, to assess treatment efficacy in younger and older patients); (3) glycemic control (HbA1c <8.0% vs. ≥8.0%, to assess the impact of baseline diabetes control); (4) ulcer duration (≤20 weeks vs. >20 weeks, to compare treatment efficacy in acute and chronic ulcers); and (5) baseline ulcer area (to study the impact of ulcer severity).

### Country

**Figure 7:**
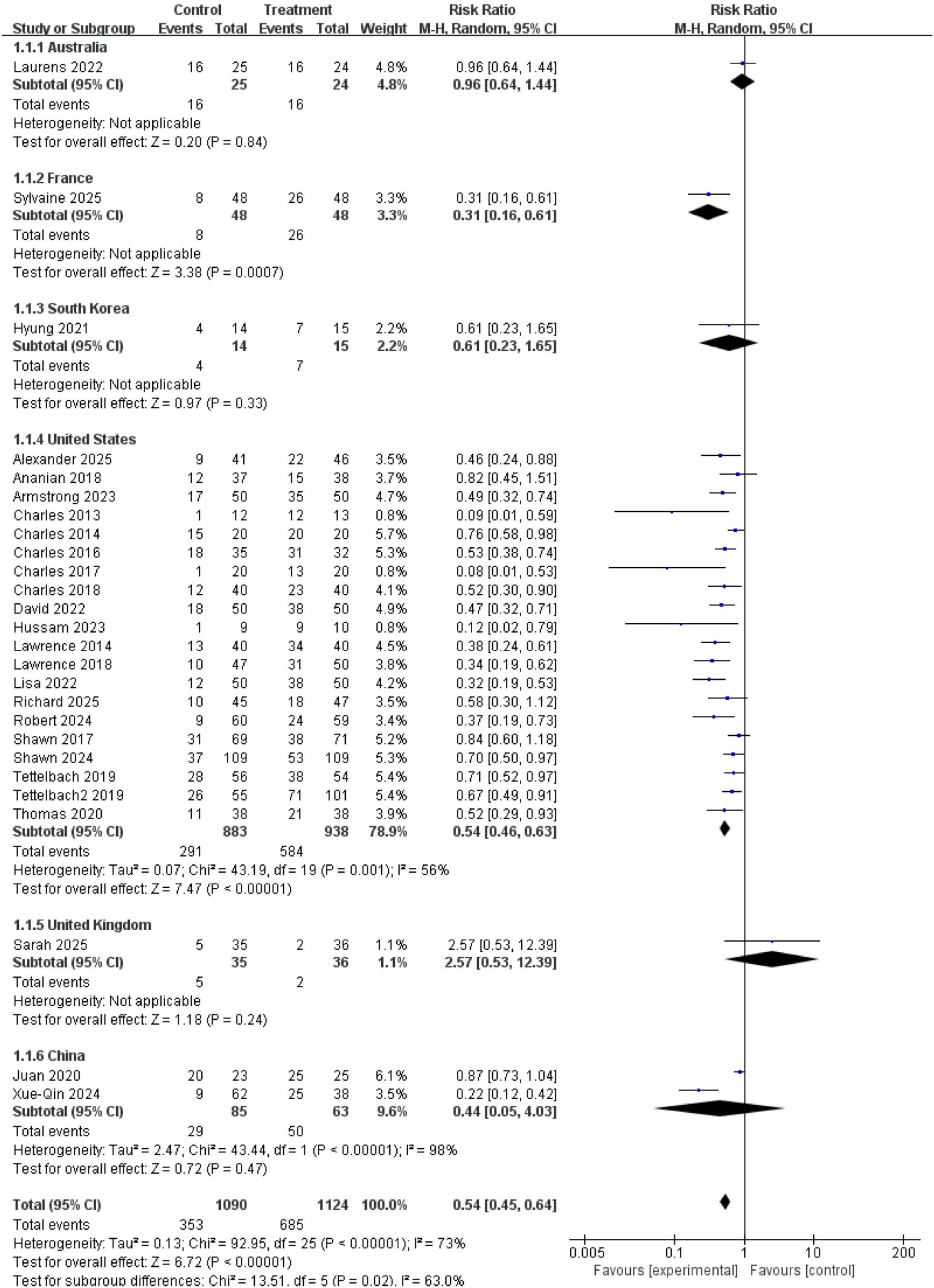
Subgroup analysis of complete healing rate by country.

Of the 26 studies included, 20 (76.92%) were conducted in the United States, constituting the main weight of the meta-analysis. The number of studies from Oceania (1), Europe (2), and Asia (3) was limited; their effect directions were consistent with the overall results, but the confidence intervals were too wide (e.g., for Europe, 95% CI 0.64-1.44). Therefore, the existing data are insufficient for a reliable quantitative comparison of treatment efficacy differences between different countries or regions. The results are shown in **Figure 7**.

### Age

≤60 years old group (RR = 0.60): The effect was very significant.

>60 years old group (RR = 0.50): The effect was also very significant, and numerically showed a greater trend of benefit.

Subgroup differences: There was no statistically significant interaction between subgroups (interaction P-value = 0.39), suggesting that age did not significantly alter the intervention effect. Both younger and older patients can benefit.

**Figure 8:**
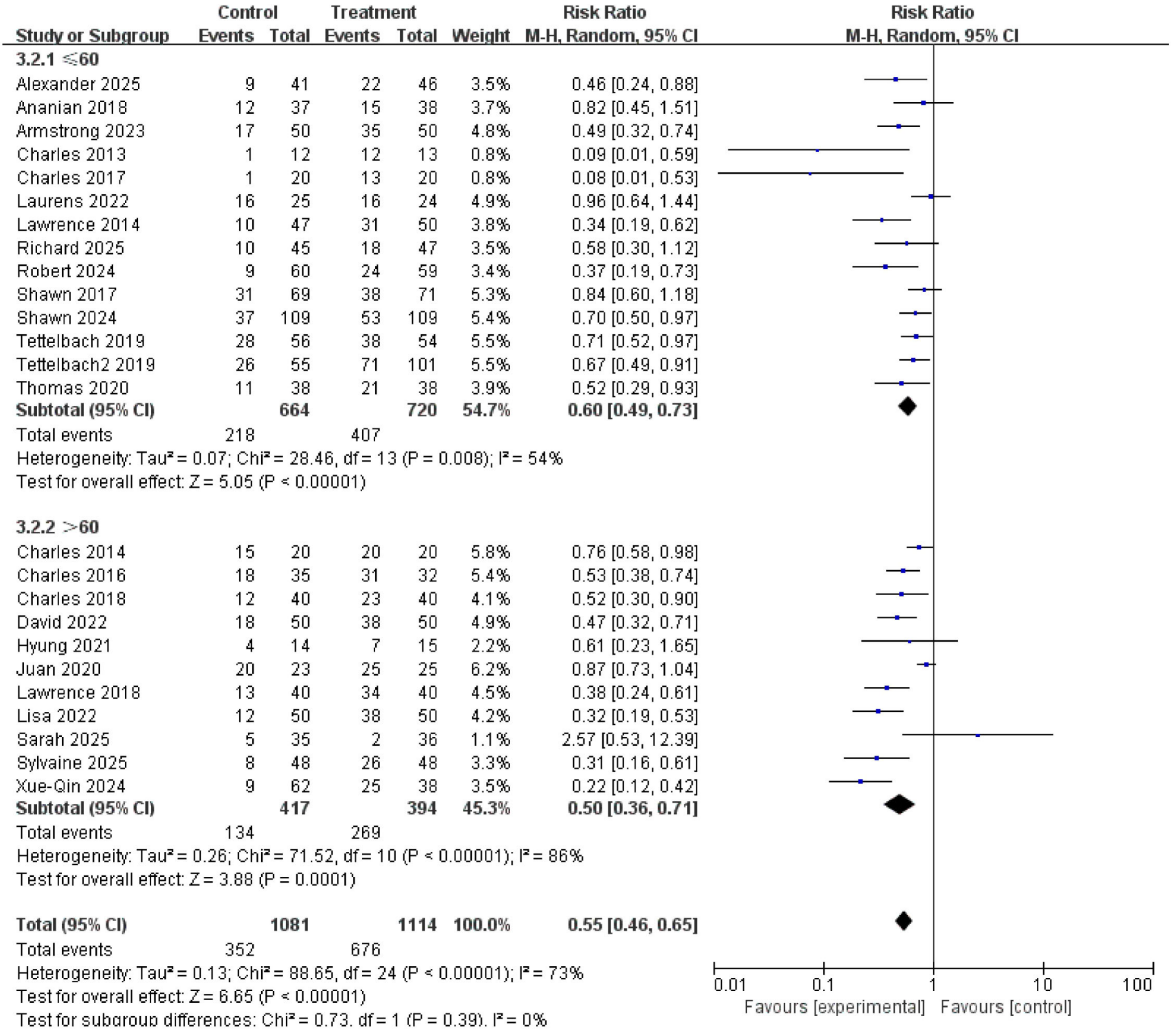
Subgroup analysis of complete healing rate by age.

Special note: In the >60 years old group, the results of the “Sarah 2025” study were anomalous (RR = 2.57, 95% CI 0.53-12.39), being the only study showing that the treatment might be harmful (23). However, its confidence interval was wide and included zero, making it statistically insignificant, possibly due to a small sample size and chance findings, and did not affect the overall conclusion for this subgroup. The results are shown in **Figure 8**.

### Glycated hemoglobin

HbA1c <8.0% group (RR = 0.46): The effect was extremely significant, showing the strongest treatment effect.

HbA1c ≥8.0% group (RR = 0.60): The effect was also very significant.

**Figure 9:**
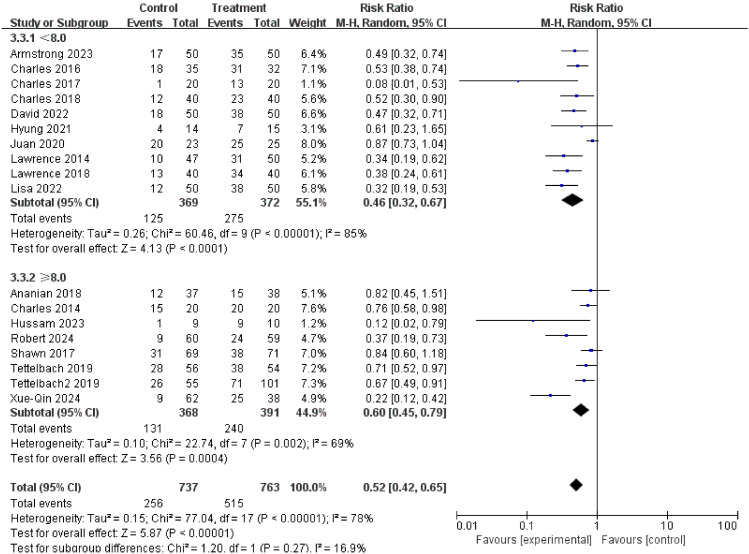
Subgroup analysis of complete healing rate by glycated hemoglobin (HbA1c).

Difference between subgroups: The statistical test was not significant (P = 0.27, I^2^ = 16.9%). However, from a clinical perspective, there was a greater trend towards benefit in patients with good glycemic control (HbA1c <8.0%), which has important clinical implications. The results are shown in **Figure 9**.

### Diabetic foot disease course

**Figure 10:**
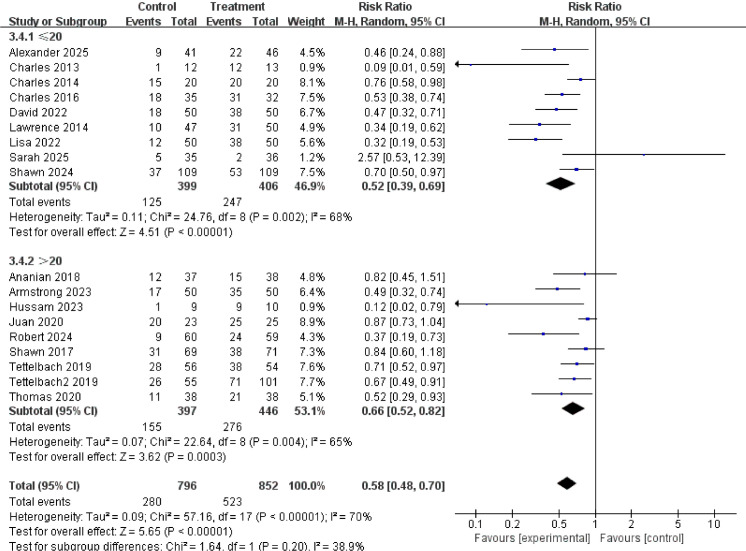
Subgroup analysis of complete healing rate by ulcer duration.

There was no significant difference in treatment efficacy between short-term (≤20 weeks) and long-term (>20 weeks) ulcers (interaction P-value = 0.85), indicating that the drug is effective for both acute and chronic wounds, which to some extent reduces the limitations of its clinical application. The results are shown in **Figure 10**.

### Ulcer area

**Figure 11:**
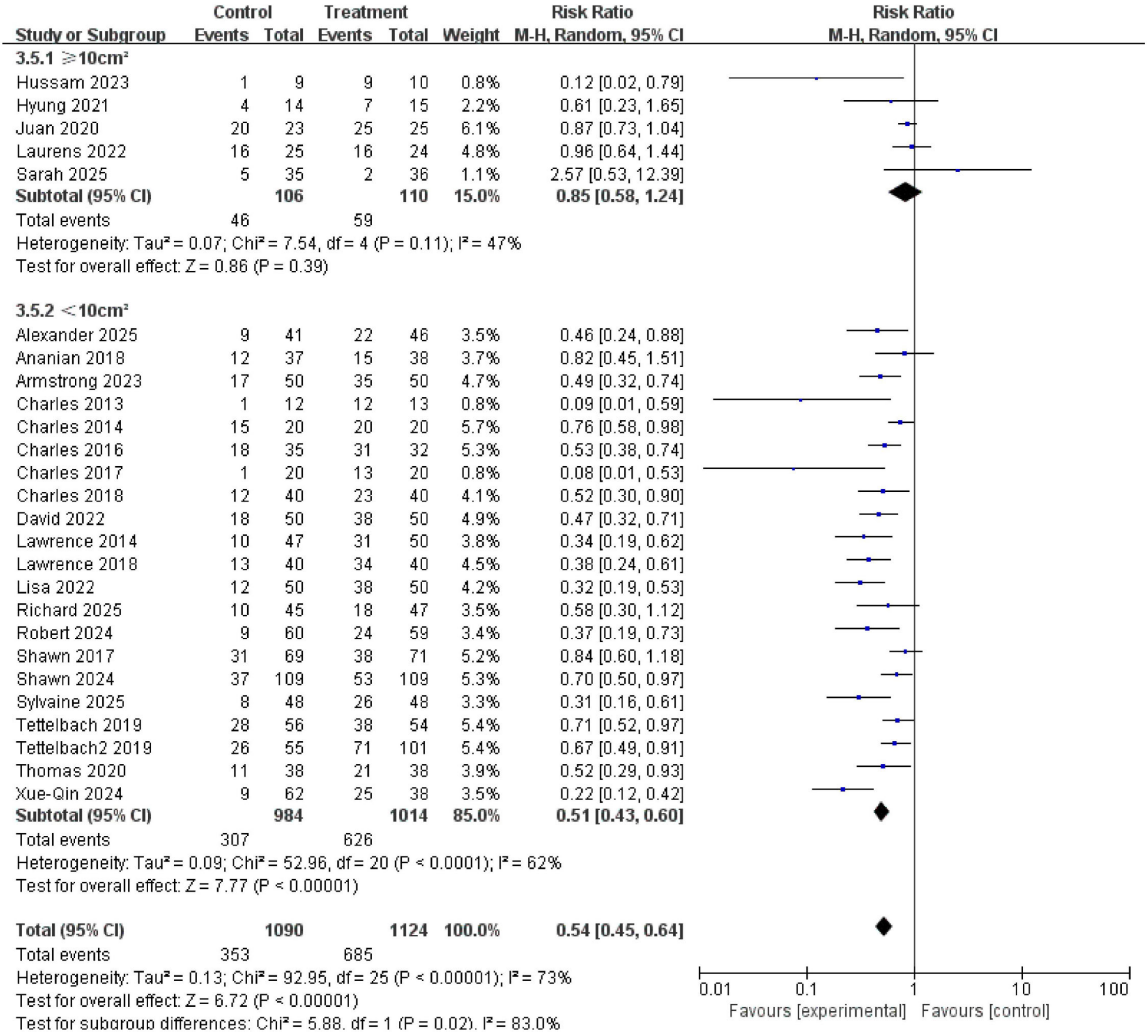
Subgroup analysis of complete healing rate by baseline ulcer area (cut-off: 10cm²).

Subgroup analysis based on baseline ulcer area was performed using 10 cm² as the cut-off value (<10 cm² vs. ≥ 10 cm²). The results indicated consistent treatment benefits across both groups, suggesting that this therapy is also suitable for patients with larger or more severe ulcers. The subgroup forest plot is shown in **Figure 11**.

### Subgroup results interpretation

Firstly, the existing data are insufficient for reliable quantitative comparisons of treatment efficacy across different countries or regions. Secondly, consistent treatment benefits were observed across all age groups (including patients over 60 years old), supporting the applicability of autologous cell therapy in elderly diabetic patients, who may have multiple comorbidities. Furthermore, the trend towards better efficacy in patients with better glycemic control (HbA1c < 8.0%) (although not statistically significant in this analysis) is consistent with known biological mechanisms – a hyperglycemic environment impairs wound healing. This emphasizes the importance of optimizing systemic diabetes management as a foundation for adjunctive cell therapy. In addition, the finding that ulcer duration did not significantly affect efficacy is encouraging, suggesting that this intervention may also be beneficial for long-standing chronic ulcers that are difficult to treat with traditional methods. However, these subgroup results should be interpreted cautiously, as they are based on exploratory analyses rather than individual patient data, limiting their ability to detect true interactions. The non-significant P-values for interactions indicate that we cannot definitively conclude that there are differences in treatment effects between subgroups. Larger prospective trials are needed to validate or refute these observed trends.

### Publication bias

**Figure 12:**
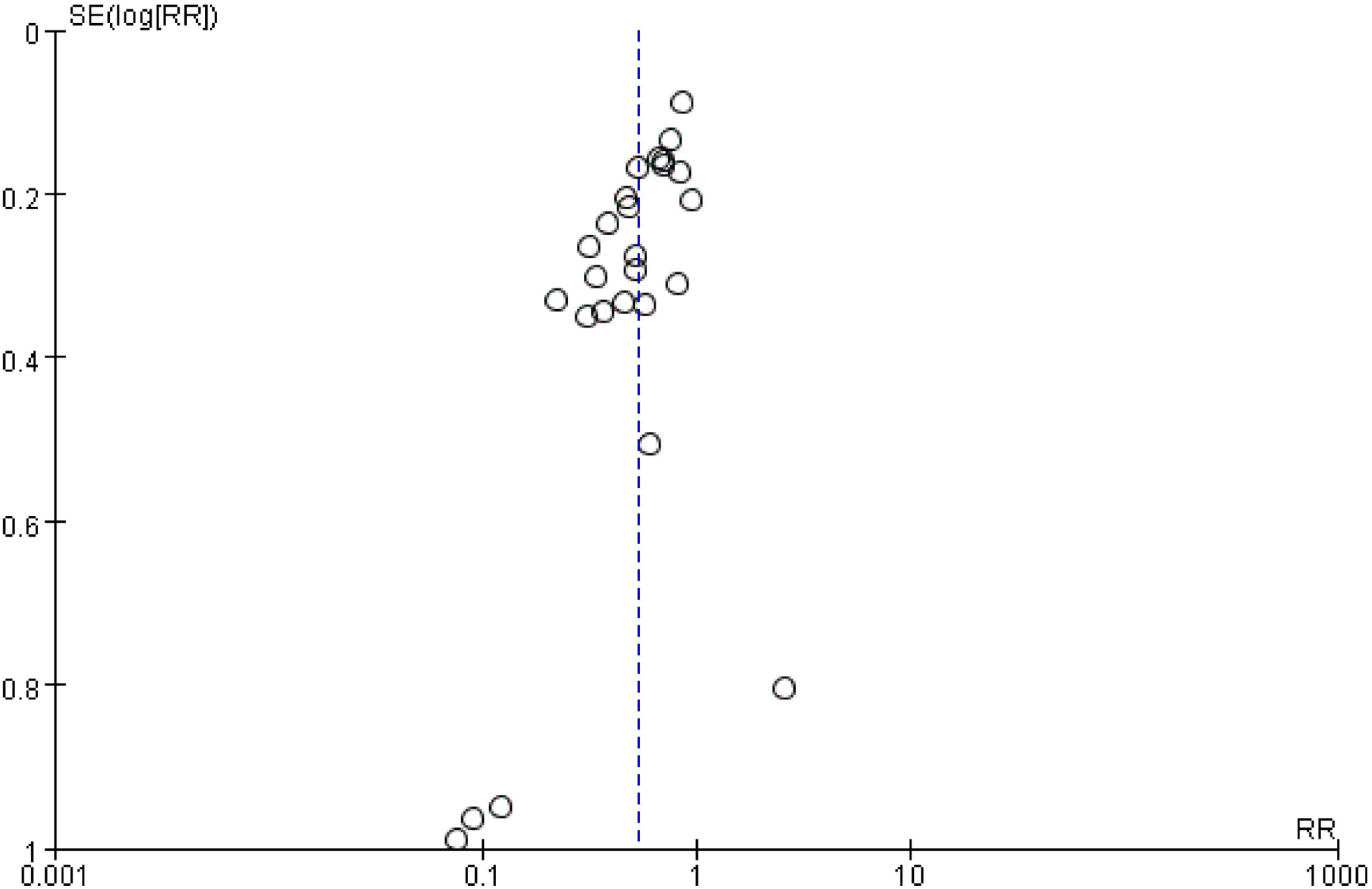
Funnel plot for publication bias.

In the complete healing rate was examined using a funnel plot. The funnel plot was symmetrical, indicating that there was no significant publication bias. The results are shown in **Figure 12**.

## Discussion

Here we present the results of a meta-analysis of 26 randomized controlled trials involving 2214 patients diagnosed with diabetic foot. This updated meta-analysis provides more comprehensive evidence on autologous cell and related products for the treatment of diabetic foot. Results showed that autologous cell and related products significantly positively impacted complete healing rates. These results suggest that autologous cells and related products may be a promising approach for treating diabetic foot. They may accelerate healing through mechanisms such as the release of growth factors, promotion of angiogenesis, and enhanced cell migration. The appendix to the guidelines notes that some trials have reported that stem cell therapy can alleviate ischemic symptoms, improve function, and prevent amputation (36, 37). Compared to previous reviews, this study is updated to 2025, covering more novel autologous cell products and providing more timely results. However, some heterogeneity exists among studies, possibly related to differences in intervention type, ulcer severity, and follow-up time. Subgroup analysis suggests that although all subgroups showed benefit, patients with good glycemic control may benefit more, highlighting the importance of comprehensive diabetes management. Furthermore, research indicates that lowering the age for Medicare benefits may benefit patients with diabetic foot ulcers (DFU) (38). Further validation with more high-quality, large-sample, long-follow-up randomized controlled trials (RCTs) is needed, along with a deeper understanding of the specific mechanisms by which autologous cells and related products treat diabetic foot. Since the included studies did not explicitly specify whether the lesions in patients with diabetic foot ulcers were neuroischemic or neuropathic, our future clinical trials should make a more careful distinction. Furthermore, since healing time impacts cost-effectiveness, we will focus more on the healing time of autologous cells and related products in treating diabetic foot, while directly comparing different cell products to analyze cost-effectiveness. Additionally, studies have found that after controlling for common risk factors, non-white patients appear to be more at risk of amputation (39). The most common location for foot ulcers is the first toe, consistent with its role as a major pressure point in the gait cycle; the location of ulceration is also an important direction for future research (40).

## Conclusion

Autologous cell therapy and related products significantly improve the complete healing rate and accelerate the healing process of diabetic foot ulcers, with good safety profiles. They do not increase the risk of amputation or death. Subgroup analysis showed that their efficacy was evident in patients of different ages and disease durations, and may be even better in patients with good glycemic control. Therefore, this study demonstrates that adding autologous cell therapy and related products to standard wound care is an effective and safe adjunctive treatment strategy, providing clinicians with a new evidence-based option for managing refractory diabetic foot ulcers. It is recommended that autologous cell therapy be considered as an adjunctive treatment option for diabetic foot ulcers in addition to standard care.

## Data Availability

The original contributions presented in the study are included in the article/supplementary material. Further inquiries can be directed to the corresponding authors.
